# Modulation of Endoplasmic Reticulum Stress by Selected Polyphenols from *Sambucus ebulus* L. Fruit

**DOI:** 10.3390/plants14172748

**Published:** 2025-09-02

**Authors:** Stoyan Stoyanov, Momchil Barbolov, Galina Yaneva, Oskan Tasinov

**Affiliations:** 1 Department of Biology, Faculty of Pharmacy, Medical University of Varna, 84 Tzar Osvoboditel Blvd., 9002 Varna, Bulgaria; stoyan.stoyanov@mu-varna.bg (S.S.); galina.yaneva@mu-varna.bg (G.Y.); 2 Department of Biochemistry, Molecular Medicine and Nutrigenomics, Faculty of Pharmacy, Medical University of Varna, 84B Tzar Osvoboditel Blvd., 9002 Varna, Bulgaria; barbolovm@gmail.com

**Keywords:** endoplasmic reticulum stress, resveratrol, chlorogenic acid, epicatechin, chrysanthemin, unfolded protein response, *Sambucus ebulus* L., dwarf elderberry

## Abstract

When misfolded or unfolded proteins accumulate in the endoplasmic reticulum (ER), ER stress occurs, which contributes to the pathogenesis of various diseases. A previous study from our research group showed that aqueous extract from the *Sambucus ebulus* L. fruit has anti-inflammatory properties, possibly by reducing ER stress. The extract was found to contain high levels of neochlorogenic acid, chlorogenic acid, idaein, epicatechin, resveratrol, and chrysanthemin. The present review summarizes the effects of these phytochemicals on ER stress. We queried the PubMed and ScienceDirect databases for primary studies discussing ER stress markers influenced by neochlorogenic acid, chlorogenic acid, idaein, epicatechin, resveratrol, and chrysanthemin. Forty-two articles were selected for review. No sufficient data were found regarding neochlorogenic acid and idaein in the context of ER stress. Other polyphenols, at low concentrations, reduce ER stress markers following exposure to stress agents in various experimental models. Interestingly, high doses of resveratrol activate pro-apoptotic signaling in cancer cell lines. A causal relationship between the polyphenols in the extract and ER stress modulation was identified. The PERK pathway was most strongly associated with the effects of the listed compounds. Although further research is needed, recent findings suggest potential therapeutic applications of these phytochemicals for conditions associated with chronic cellular stress.

## 1. Introduction

The endoplasmic reticulum is an organelle present only in eukaryotic cells, where it performs various functions, including protein synthesis, post-translational protein modification, participation in lipid metabolism, calcium ion storage, and glycogen production [1]. The lumen of this organelle contains chaperones and enzymes necessary for the proper folding of newly synthesized secretory and membrane proteins, such as PDI (protein disulfide isomerase), GRP78/BiP (glucose-regulated protein 78/binding immunoglobulin protein), GRP94 (glucose-regulated protein 94), etc. [2]. Endoplasmic reticulum (ER) stress occurs when misfolded or unfolded proteins accumulate within the ER lumen. It can be caused by a variety of factors, including increased demand for protein synthesis, genetic mutations, oxidative stress, and more [3]. There are special signaling pathways in cells, known as the unfolded protein response (UPR), whose purpose is to rid the cell of ER stress and restore homeostasis.

(1) IRE1 (Inositol-requiring enzyme 1) signaling cascade—IRE1 is associated with the BiP chaperone and remains inactive under normal conditions. Upon accumulation of misfolded proteins, BiP dissociates from the receptor and binds to these proteins. The now-activated IRE1 dimerizes, undergoes autophosphorylation, and initiates splicing of XBP1 (X-box binding protein 1) mRNA by cutting away an intron. This generates XBP1s mRNA, which encodes the transcription factor XBP1s, which translocates into the nucleus and regulates the activity of important genes encoding chaperones, ERAD (endoplasmic reticulum-associated degradation) components, enzymes involved in lipid biosynthesis, and oxidative phosphorylation. Additionally, IRE1 degrades selected mRNAs through a mechanism known as regulated IRE1-dependent decay (RIDD) to lower protein production load. Under conditions of severe ER stress, activated IRE1 binds to TRAF2 (TNF receptor-associated factor 2), which activates ASK1 (apoptosis signal-regulating kinase 1). ASK1 then phosphorylates JNK (c-Jun N-terminal kinase), leading to programmed cell death and regulation of genes involved in inflammation, differentiation, and redox homeostasis. Moreover, TRAF2 activates IκB kinase (IKK), which phosphorylates IκB (inhibitor of NF-kB), resulting in the release of NF-κB. NF-κB translocates to the nucleus, where it regulates the expression of genes related to inflammation, immune response, and cell proliferation [2,4,5,6].

(2) ATF6 (Activating Transcription Factor 6) signaling cascade—when misfolded proteins accumulate, BiP dissociates from the ATF6 receptor, allowing ATF6 to translocate to the Golgi apparatus. There, it is cleaved by the proteases S1P (site-1 protease) and S2P (site-2 protease), producing its active form as a transcription factor. ATF6 then enters the nucleus and promotes the expression of genes encoding chaperones, ERAD components, and XBP1 [5,7].

(3) PERK (Protein kinase RNA-like ER kinase) signaling cascade—under ER stress, BiP is released from PERK, leading to its autophosphorylation and activation. Activated PERK phosphorylates the α subunit of eukaryotic initiation factor 2 (eIF2α), which suppresses general protein synthesis while promoting the selective translation of ATF4. ATF4 leads to increased expression of genes involved in chaperone production, antioxidant response, amino acid transporters, and proteins necessary for autophagy. If the stress is too severe, ATF4 induces the expression of C/EBP-homologous protein (CHOP), also known as GADD153, a pro-apoptotic factor [2,5,8].

**Figure 1:**
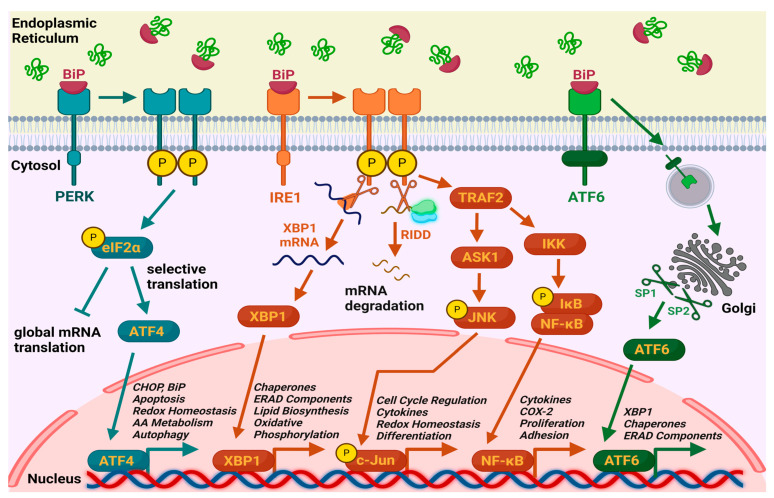
Unfolded protein response (UPR) pathways. Schematic generated using BioRender. Momchil Barbolov (2025), https://app.biorender.com/i-668cf1bc2e623fff293e1ca0-untitled (accessed on 24 July 2025).

This can be achieved through decreased protein synthesis, increased production of folding-facilitating molecules, or enhanced degradation of misfolded proteins. However, if homeostasis cannot be restored, the UPR may ultimately trigger apoptosis (Figure 1) [5]. Chronic inflammation can both induce and be a result of ER stress. Activation of the PERK and IRE1 pathways leads to stimulation of inflammatory signaling [9,10].

ER stress is involved in the pathogenesis of various diseases, such as neoplastic, metabolic, and neurodegenerative disorders. The regulation of this adaptive response is an important mechanism guaranteeing the survival and growth of tumor cells [11]. They use mechanisms such as an increase in GRP78 chaperone levels and activation of the XBP1 cascade to survive in unfavorable conditions [12,13,14]. Hepatitis C virus successfully suppresses the IRE1-XBP1 pathway [15]. The accumulation of the large surface protein of Hepatitis B virus also leads to ER stress [16]. Ethanol induces CHOP expression and triggers hepatocyte apoptosis [17]. Cellular stress promotes the activation of the transcription factor SREBP-1c, a key regulator of lipogenesis in the liver, favoring the development of non-alcoholic fatty liver disease (NAFLD) [18]. ER stress also contributes to the development of type 2 diabetes and obesity by impairing insulin signaling [19]. CHOP-mediated apoptosis of foam cells in the vessels of the heart contributes to the rupture of atherosclerotic plaques and the onset of acute coronary syndrome [20]. There is a link between ER stress and some neurological diseases as well. Mutations associated with familial Alzheimer’s disease and juvenile Parkinson’s disease increase sensitivity to ER stress and disrupt cellular defense mechanisms [21,22].

Polyphenols are found in a wide range of fruits, vegetables, drinks, and cereals. They provide protection from ultraviolet radiation and pathogens, and partly determine flavor profiles. Their potential health benefits have been extensively studied in recent decades. Polyphenols are categorized into four main classes: phenolic acids, flavonoids, stilbenes, and lignans. Flavonoids are further divided into six subclasses based on their specific structural characteristics: flavones, flavanones, flavonols, flavanols, anthocyanins, and isoflavones [23].

*Sambucus ebulus* L., also known as dwarf elder or danewort, is a perennial herbaceous plant [24]. Its stem is 100–200 cm long and the leaves are opposite and pinnate, reaching up to 30 cm in length. The stem ends in a corymb of white or pink flowers. The fruit is small, dark blue to violet, with three seeds. The blooming period is from May to August, with fruit maturation occurring from August to September [25]. *S. ebulus* is found in Southwest Asia, Northwest Africa, and Southern and Central Europe, where it is present in the folk medicine of many countries [26]. The fruits are the most commonly utilized part of the plant, as they contain the highest concentration of polyphenolic compounds. They have traditionally been used in the form of jam for the treatment of tuberculosis and hemorrhoids, fresh or in the form of bath decoctions for the treatment of rheumatoid arthritis, as well as in syrup form for appetite-stimulating and tonic action [25,27,28,29]. The phytochemical composition of *S. ebulus* includes various compounds that play an important role in its biological properties and medicinal effects, such as iridoid glycosides, flavonoids, phenols, phytosterols, anthocyanins, caffeic acid derivatives, triterpenes, tannins, ribosome-inactivating proteins, and lectins [28,30,31,32,33,34,35]. Compared to other members of the *Sambucus* genus, such as *Sambucus nigra* and *Sambucus cerulea*, *Sambucus ebulus* has a higher content of phenolic acids, flavonols, and proanthocyanidins [36]. There are a number of studies evaluating the potential of elderberry extracts to inhibit oxidative stress, based on their rich polyphenol and vitamin content [37,38,39,40]. Additionally, the plant has been reported to exhibit anti-inflammatory properties [41,42,43,44,45], which can be attributed to certain compounds found in the herb, such as anthocyanins, quercetin, resveratrol, and chlorogenic acid [46,47,48,49]. Anticancer, antiviral, antibacterial, antifungal, and antiparasitic effects have also been described for *S. ebulus* extracts [28].

**Figure 2:**
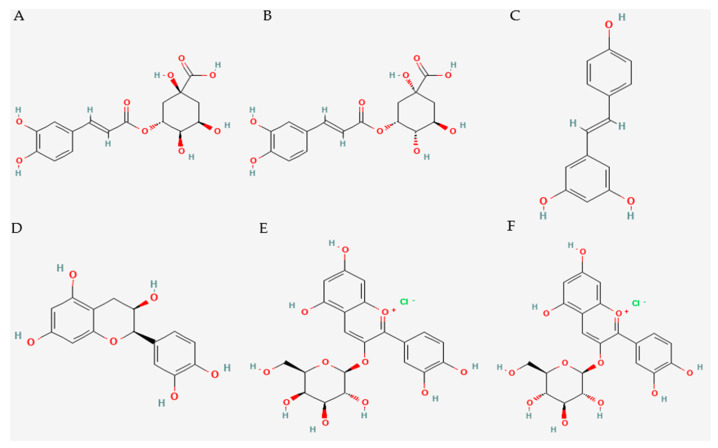
Chemical structure of chlorogenic acid (**A**), neochlorogenic acid (**B**), resveratrol (**C**), epicatechin (**D**), cyanidin-3-O-galactoside (**E**), and cyanidin-3-O-glucoside (**F**). Available from https://pubchem.ncbi.nlm.nih.gov/ (accessed on 12 August 2025).

In recent years, a number of studies have shown that plant extracts can modulate ER stress through targeted effects on UPR pathways [50,51,52,53]. Our research group recently analyzed the phytochemical composition and biological activity of an aqueous-alcoholic extract of *S. ebulus* fruits. The results of the chemical analysis, presented as mean ± standard deviation, revealed a high content of the following compounds: neochlorogenic acid (906.08 ± 32.84 µg/mL), chlorogenic acid (567.06 ± 20.55 µg/mL), cyanidin-3-O-galactoside or idaein (382.15 ± 13.19 µg/mL), epicatechin (322.37 ± 11.75 µg/mL), resveratrol (51.92. ± 1.94 µg/mL), and cyanidin-3-O-glucoside or chrysanthemin (31.07 ± 1.10 µg/mL) (Figure 2). Using the J774A.1 macrophage cell line, the study demonstrated significant anti-inflammatory properties of the extract, accompanied by a reduction in key endoplasmic reticulum stress markers, including peIF2α, ATF6, and CHOP, under conditions of LPS-induced inflammatory activation. The reported decrease in the expression of these proteins suggests that modulation of ER stress is a likely additional mechanism by which the extract exerts its anti-inflammatory effects [44]. However, the specific phytochemical components responsible for the modulation of ER stress were not identified in the cited study. The present review aims to summarize the effects of the most prevalent polyphenolic compounds identified in the extract on mechanisms related to endoplasmic reticulum stress, based on available data from scientific literature. Thus, the potential causal relationship between the polyphenols contained in the *S. ebulus* extract and the observed biological effects can be elucidated. Furthermore, the review outlines key limitations in the current literature and provides recommendations for future studies.

## 2. Materials and Methods

### 2.1. Search Strategy

We applied the “Advanced search” option in two databases, Pubmed and ScienceDirect, where we used the following keywords and Boolean operators: “Neochlorogenic acid” and “ER stress”, “Chlorogenic acid” and “ER stress”, “Resveratrol” and “ER stress”, “Cyanidin-3-o-glucoside” OR “Chrysanthemin” and “ER stress”, “Cyanidin-3-o-galactoside” OR “Idaein” and “ER stress”, “Epicatechin” and “ER stress”. We refined the results by adding supplementary filters to ensure relevance and quality:

- Text availability—free full texts
- Publication date—from 2000/1/1 to 2025/5/1
- Article type—research articles
- Subject area—Biochemistry, Genetics, and Molecular Biology
- Access type—open access and open archive

### 2.2. Inclusion and Exclusion Criteria

The review included in vivo or in vitro studies that focused on the effects of the individual polyphenolic compounds (resveratrol, cyanidin-3-o-glucoside, cyanidin-3-o-galactoside, epicatechin, neochlorogenic acid, and chlorogenic acid) on ER stress. Full text articles discussing markers and pathways, affected by these phytochemicals, and providing sufficient experimental details (e.g., dosage, model system) for evaluation were also defined as inclusion criteria.

Studies were excluded if they examine plant extracts or analogues of the specific isolated compounds. Articles that did not evaluate ER stress key markers or molecular pathways were not considered. Records limited to anti-inflammatory, metabolic, antioxidant, or other biological effects of ingredients, without any relation to ER stress, were also excluded.

### 2.3. Statistical Analyses and Presentation

MS Excel 2016 was used for statistical processing and graphical presentation of the collected data. BioRender was used to build a schematic for the ER stress signaling pathways.

## 3. Results

### 3.1. Study Selection

**Figure 3:**
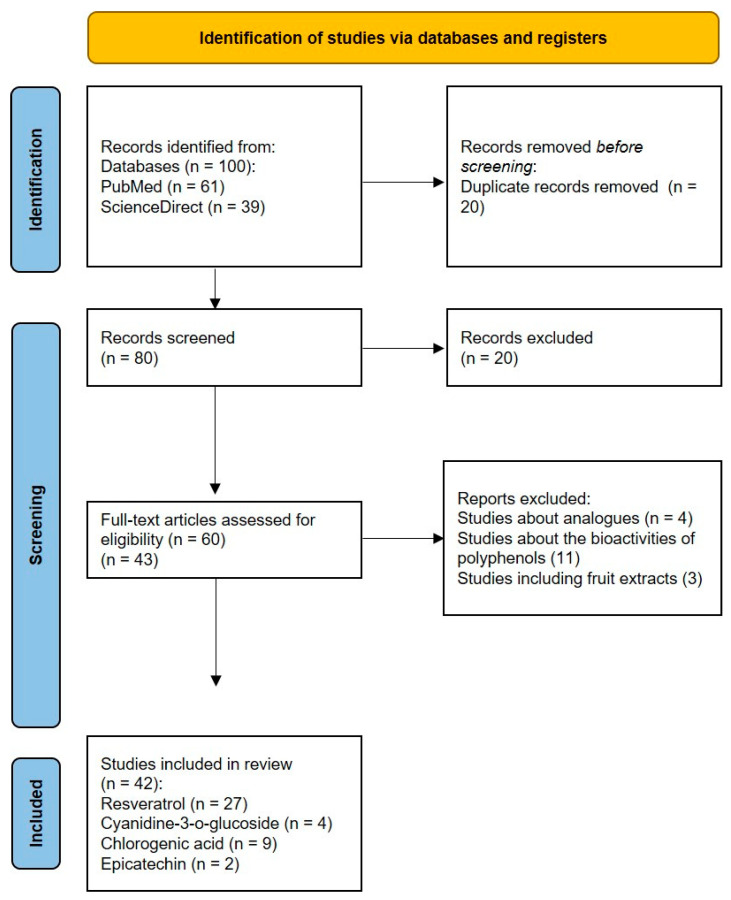
PRISMA flow diagram of the study selection process.

We were able to find 100 records from the PubMed and ScienceDirect databases. The study selection process followed the PRISMA flowchart (Figure 3). First, twenty duplicate records were removed. After screening the remaining 80 articles, 20 titles or abstracts did not meet the inclusion and exclusion criteria, and were therefore excluded. Sixty full-text articles were assessed for eligibility. Studies discussing biological properties of plant extracts, containing the listed phytochemicals or their analogues, were not considered. Ultimately, 42 articles underwent literary analysis.

### 3.2. Presentation of the Results

Available experimental evidence on the effects of the selected compounds on UPR signaling was found for only four of the six phytochemicals listed in the inclusion criteria—resveratrol, chlorogenic acid, epicatechin, and cyanidin-3-o-glucoside.

#### 3.2.1. Modulation of ER Stress by Resveratrol

The influence of resveratrol on endoplasmic reticulum stress has been widely investigated by multiple groups. Analysis of the studies revealed that resveratrol modulates the ER stress in different ways depending on the dose and the type of experimental model used, as presented in Table 1. Under conditions of induced ER stress, and when administered at low doses, resveratrol suppresses UPR signaling and reduces the expression of key markers such as GRP78, GRP94, CHOP, p-PERK, IRE1, p-eIF2α, and ATF6, thus exhibiting a protective effect [54,55,56,57,58,59,60,61,62,63,64]. Conversely, at higher doses—particularly in tumor cell lines—resveratrol enhances ER stress and apoptosis, which is an indication of its cytotoxic action [60,65,66,67,68,69]. The data show that this polyphenolic compound primarily affects the PERK and IRE1 signaling cascades and modulates the expression of proteins and chaperones involved in the proper folding of newly synthesized polypeptide chains [56,64,65,66,70,71,72,73,74].

#### 3.2.2. Modulation of ERS by Cyanidin-3-o-Glucoside, Chlorogenic Acid, and Epicatechin

According to the literature, in experimental models of induced ER stress, cyanidin-3-o-glycoside decreases the expression of GRP78, p-PERK, p-eIF2α, ATF4, CHOP, IRE1, XBP1, and ATF6, which indicates its potential to suppress UPR signaling. A similar effect is observed for chlorogenic acid, but its impact on the IRE1 pathway is less characterized. Although data on epicatechin are scarce, it also has the ability to regulate protein homeostasis by acting on IRE1 and PERK signaling pathways. These polyphenols have a protective effect at low doses, similar to the effects of resveratrol (Table 2).

## 4. Discussion

Our study investigates and summarizes the known effects of specific polyphenols on endoplasmic reticulum (ER) stress, a process implicated in the development of a wide range of diseases, as mentioned above. We selected neochlorogenic acid, chlorogenic acid, idaein, epicatechin, resveratrol, and chrysanthemin for this review, based on two factors. First, our previous study demonstrated ER stress-modulating activity of an aqueous extract from *S. ebulus* fruit. We assume that this activity is mainly due to the compounds that are present in the highest concentrations in the extract [44]. Second, other reports identified these compounds as quantitatively dominant in the plant’s phytoprofile or emphasized its high phenolic content [28,36,96]. In addition, for the selected polyphenols there are published data suggesting a potential role in modulating endoplasmic reticulum stress, except for idaein and neochlorogenic acid. However, no systematic review has yet examined the effects of the phytochemicals found in *S. ebulus* extracts on ER stress.

In general, the four examined polyphenols act on one or more branches of UPR signaling. While some studies do not specify the exact branch affected, altered expression levels of certain molecules suggest the pathway involved. Considering Table 1 and Table 2, the polyphenols most frequently influence the PERK signaling cascade, along with a reduction in CHOP expression, a critical mediator of ER stress-induced apoptosis. Since all four polyphenols affect PERK and IRE1, this may explain the observed anti-inflammatory properties of the dwarf elderberry extract, as these pathways can directly activate pro-inflammatory cascades [10]. Data from two independent studies suggest that epicatechin does not significantly modulate the ATF6 signaling pathway. However, there are some key methodological differences—one study used antibodies against cleaved ATF6 as part of the protocol, while the other did not include such antibodies [85,86]. Thus, the influence of this polyphenol on the ATF6 branch of the UPR is still unexplored, highlighting the need for further investigation. Experimental studies are also needed to address the current lack of data on the effects of neochlorogenic acid and idaein on ER stress.

The precise molecular mechanisms by which these phenolic compounds exert their effects on ER stress are not fully understood. It is known, for instance, that the activation of sirtuin 1 (SIRT1) is key to resveratrol’s activity. SIRT1 is an NAD+-dependent protein deacetylase that modulates the activity of molecules involved in the UPR pathway [54,59,60,76]. SIRT1 stimulation has also been reported for cyanidin-3-O-glucoside [97]. Resveratrol’s protective effect under induced endoplasmic reticulum (ER) stress may also be mediated by clusterin, a molecular chaperone induced by stress, although its connection to SIRT1 remains unclear [59]. Moreover, a mechanism independent of SIRT1 has been reported by Graham et al., who suggest that resveratrol stimulates the enzyme protein phosphatase 1α (PP1α), which in turn inhibits protein kinase B (Akt), a modulator of protein synthesis and endoplasmic reticulum stress [74]. Furthermore, in prostate cancer cell lines, resveratrol has also been shown to deplete calcium stores in the ER and suppress the store-operated Ca^2+^ entry (SOCE) mechanism, ultimately leading to ER stress and cell death [68]. Further research should focus on elucidating the mechanisms by which phytochemicals modulate the expression of key markers of UPR signaling, especially in the case of epicatechin and chlorogenic acid.

The effects of polyphenols on endoplasmic reticulum stress in various in vivo and in vitro models suggest that these substances could be used as therapeutic agents. Consequently, plant sources containing these compounds may also be of interest as functional foods. For example, resveratrol, a stilbene, is found in grapes, red wine, berries, and peanuts, while chrysanthemin, an antocyanin, is present in berries, black beans, and black rice [98,99,100,101]. Epicatechin is abundant in green tea [102]. Coffee, apples, berries, and potatoes are sources of chlorogenic acid, a type of phenolic acid [103]. When considering the effects of polyphenols, it is important to take into account their bioavailability. Because of the intense biotransformation of resveratrol into glucuronides and sulfates in the intestines and liver, the oral bioavailability of resveratrol is less than 1% [104]. Upon oral administration, the stilbene first comes into contact with these organs, so we may assume that it can regulate ER stress locally in them. Some of the biological effects caused by resveratrol may be either directly because of its metabolites or the resveratrol released into the tissues by them [105,106]. There is no adequate pharmacokinetic data for chlorogenic acid due to its limited bioavailability [107]. Most of the chlorogenic acid is converted by intestinal microflora into smaller metabolites that enter the bloodstream. A small portion of the acid is absorbed intact [107,108,109]. Therefore, the observed in vivo effects are likely to be the result of chlorogenic acid and its active metabolites. Cyanidin-3-O-glucoside and its metabolites have high absorption, bioavailability, and biological effects [101,110,111]. Epicatechin is partially absorbed and present in the form of various conjugates in plasma [112]. Epicatechin conjugates reach tissues through the bloodstream. However, no studies have been conducted to date on their biological activity [113,114]. According to the data presented, cyanidin-3-O-glucoside has the highest therapeutic potential. A pharmacodynamic characterization of epicatechin conjugates is necessary because without it, an accurate assessment may be difficult. Chlorogenic acid has significant potential, but it depends on individual variations in intestinal microbial metabolism. Despite its low oral bioavailability, resveratrol remains valuable because its metabolites may also contribute to its effects. There are many promising ways to increase the bioavailability of stilbenoids and chlorogenic acid, such as using bioenhancers, nanocapsulation in lipid nanocarriers or liposomes, etc., but further research is needed in this area [107,109,115,116].

Models of induced ER stress clearly show that, when administered at low doses, chlorogenic acid, chrysanthemin, and resveratrol exert a protective effect by dose-dependently downregulating the expression of molecules associated with UPR signaling or apoptosis. In a pathological context, this protective function may slow disease progression and alleviate symptoms. As shown in Table 1, high doses of resveratrol, on the other hand, induce endoplasmic reticulum stress and activate apoptotic mechanisms, especially in tumor cell lines, which may be therapeutically advantageous. Thus, resveratrol exerts a complex dual effect on ER stress. The contradictory findings regarding the dual effect of resveratrol on ER stress and the expression of the proapoptotic molecule CHOP also require discussion and cannot yet be explained entirely by the type of experimental model and the dose applied. The observed effects may be antagonized or enhanced by the presence of other biologically active compounds in the plant mixtures, or modified by methodological differences in measurements (e.g., whether CHOP or CHOP mRNA is measured). Some other factors may also contribute to the conflicting conclusions. As explained above, some of the biological effects of resveratrol may be due either to resveratrol itself or to its metabolites. It is possible that some metabolites exert different activities on SIRT1 and other targets in the cell, which may have different effects (e.g., decreased or increased CHOP production) in different in vivo and in vitro models. SIRT1 activity in certain cell types may also influence the effects of resveratrol. A study by Luo et al. (2023) clearly highlighted the role of SIRT1 as a mediator of resveratrol’s effects, as inhibition of this enzyme abolished the compound’s protective action against bupivacaine-induced neurotoxicity in PC12 cells [64]. We can assume that if SIRT1 expression or activity is high in cells, resveratrol will likely induce cellular protection and a decrease in CHOP. According to the literature data, SIRT1 expression in tumor cells varies. Some cells show increased expression, which suggests that resveratrol may enhance tumor progression and survival [117,118,119]. However, we observed a pronounced antiproliferative effect of resveratrol. This effect may be due to the complex action of multiple factors, including the tumor’s genetic profile, the activity of other signaling pathways, metabolic status of the cell, and the interaction with molecules that can reverse the expected effect, leading to the suppression of tumor growth. Additionally, resveratrol has SIRT1-independent effects that may affect cellular homeostasis and induce stress. Low SIRT1 expression promotes prostatic intraepithelial neoplasia development. However, in prostate cancer cell lines, resveratrol reduces endoplasmic reticulum calcium storage, which activates programmed cell death [68,119,120]. Whether resveratrol restores normal cellular homeostasis or causes stress on the endoplasmic reticulum may depend on its ability to induce autophagy. Resveratrol stimulates autophagy through SIRT1 or AMPK (AMP-activated protein kinase) signaling, which clears misfolded proteins from the cell. In cells with blocked autophagy, this protective mechanism will be limited or absent [121,122,123].

Some studies further show that combining resveratrol with other chemicals stimulates ER stress in tumor cells, even at lower concentrations [70,74,78]. A study by Wang et al. (2011) reported a paradoxical finding: in multiple myeloma cell lines, resveratrol activated the unfolded protein response (UPR) via the IRE1 signaling cascade, promoting XBP1 splicing, while simultaneously suppressing the activity of XBP1s as a transcriptional factor through SIRT1 [66]. This suggests that resveratrol may stimulate stress while hindering the cell’s ability to adapt. In another study, low doses of resveratrol were shown to reduce the levels of p-PERK and ATF4, but activated the ATF6 pathway [60]. This suggests that apoptosis-promoting pathways are suppressed, while activation of ATF6 may play a protective role, as it is responsible for the production of chaperones and ERAD factors. At high doses, the effect was reversed.

The interpretation of available data has several limitations:

- Insufficient information on the effects of some polyphenols across all UPR branches.
- Heterogeneity in experimental models limits direct comparisons and generalization.
- Dose variation and dual effect of resveratrol on ER stress—determining the precise therapeutic dose and anticipating potential side effects is challenging.
- Lack of clinical trials and data on long-term effects of polyphenol intake—it is difficult to determine the therapeutic potential of polyphenols, as well as to assess the long-term safety and efficacy of these compounds.
- Although the results highlight the significant contribution of the polyphenols found at the highest concentrations in the aqueous dwarf elder extract, the possible biological activity of other present phytochemicals in lower amounts cannot be excluded.

Other future research should focus on pro-apoptotic effects of other polyphenols, given that high-dose resveratrol has been found to have the same effect in cancer cell lines due to the malfunction of ER. More studies investigating tissue-specific and dose-dependent effects of individual polyphenols or combinations in different disease models are essential. Clinical trials, additional research on bioavailability, and long-term safety studies are also needed to determine their potential as therapeutic agents.

## 5. Conclusions

The review identifies a possible link between the most represented polyphenols in the dwarf elder fruit aqueous extract and the modulation of ER stress. Among these, only four of the most abundant polyphenols—resveratrol, chrysanthemin, chlorogenic acid, and epicatechin—have been found to influence UPR pathways, with a predominant effect on the PERK/eIF2α/ATF4/CHOP pathway. The data indicate that these phytochemicals reduce ER stress markers in a dose-dependent manner across various in vivo and in vitro experimental models. Notably, resveratrol is the only compound among them that can amplify ER stress and provoke programmed cell death at high doses. This dual role underscores the potential of dwarf elderberry extract as a remedy for conditions associated with chronic cellular stress. Nevertheless, further research is essential to elucidate the molecular mechanisms by which phytochemicals act, determine their optimal dosages, identify potential side effects, and explore their synergistic interactions with other phytochemicals and drugs.

## Figures and Tables

**Table 1:** Modulation of ER stress markers by resveratrol.

Author(s)	Year	Experimental Model	Altered Expression or Activity of ER Stress Markers	Dosage
Chinta et al. [65]	2009	In vitro (dopaminergic N27 cells)	↑cleavage of caspases 7 and 3 ↑GRP78 expression ↑GRP94 expression ↑CHOP expression ↑p-eIF2α expression	50–250 μM
Wang et al. [66]	2011	In vitro (multiple myeloma cell lines ANBL-6, OPM2, MM.15)	↑JNK phosphorylation ↑CHOP expression ↑XBP1 mRNA splicing ↓transcription of XBP1s	100 μM
Li et al. [54]	2011	In vitro (tunicamycin-induced ER stress in HepG2 cells)	↓XBP1 mRNA splicing ↓GRP78 expression ↓CHOP expression	10 μM
Rojas et al. [67]	2014	In vitro (palmitate-induced ER stress in HepG2 cells)	↑XBP1 mRNA splicing ↑CHOP expression	100 μM
Zhang et al. [55]	2015	In vitro (cigarette smoke extract-induced apoptosis in cultured human bronchial epithelial cells)	↓CHOP expression ↓caspases 3 and 4 expression	20 µmol/L
Pan et al. [73]	2015	In vivo (high-fat diet-fed rats)	↓ATF4 expression ↓GRP78 expression ↓CHOP expression ↓GRP78 expression ↓p-PERK expression	100 mg/kg
Graham et al. [74]	2016	In vitro (2-deoxy-D-glucose inhibition of glycolysis in neuroblastoma cells)	↑CHOP expression ↓GRP78 expression ↓GRP94 expression	10 μM
Lin et al. [56]	2016	In vitro (neonatal rat cardiomyocytes)	↓GRP78 expression ↓GRP94 expression ↓CHOP expression	50 μM
Cheng et al. [57]	2016	In vitro (tunicamycin and Aβ25-35 induced ER stress in SH-SYSY cells)	↓GRP78 expression ↓CHOP expression ↓p-eIF2α expression	25 μM
Selvaraj et al. [68]	2016	In vitro (PC3 and DU145 prostate cancer cell lines)	↑CHOP expression	100 μM
Ding et al. [75]	2017	In vivo (high-fat diet-fed rats)	↓GRP78 expression ↓CHOP expression	200 mg/kg
Yan et al. [58]	2018	In vitro (tunicamycin-induced ER stress in neuronal HT22 cells)	↓GRP78 expression ↓CHOP expression ↓caspase 12 expression	50 μM
Heo et al. [69]	2018	In vitro (A375SM melanoma cells)	↑p-eIF2α expression ↑CHOP expression	10 μM
Ardid-Ruiz et al. [76]	2018	In vivo (diet induced obesity in rats)	↓XBP1s expression	200 mg/kg
Wang et al. [72]	2018	In vivo (surgical mice model)	↓GRP78 expression ↓XBP1 expression ↓PERK expression ↓IRE1 expression	100 mg/kg
Lee et al. [59]	2019	In vitro (tunicamycin-induced ER stress in HepG2 cells)	↓PERK expression ↓IRE1 expression ↓CHOP expression ↑ERAD factors expression	10, 50, and 100 μM
Zhao et al. [60]	2019	In vivo (high-fat diet-fed mice) In vitro (palmitic acid-induced insulin-resistant HepG2 cells)	in vivo model—↓p-PERK expression and ↓ATF4 expression in vitro model—↑p-PERK expression, ↑ATF4 expression, ↓ATF6 expression (at 50 and 100 μM) and ↓p-PERK expression, ↓ATF4 expression, ↑ATF6 expression (at 20 μM)	60 mg/kg (in vivo model) 20, 50, and 100 μM (in vitro model)
Lu et al. [77]	2019	In vitro (fibroblast-like synoviocytes treated with H_2_O_2_)	↑CHOP expression ↑caspase 12 and caspase 3 expression	50, 100, 200, and 400 μM
Pan et al. [71]	2019	In vivo (induced vasculitic peripheral neuropathy by ischemia–reperfusion in rats)	↓p-PERK expression ↓p-IRE1 expression ↓ATF6 expression	20 and 40 mg/kg
Zhang et al. [61]	2020	In vivo (db/db mice) In vitro (high glucose induced ER stress in NRK-52E cells)	↓GRP78 expression ↓CHOP expression ↓caspase 12 expression	20 μM (in vitro model) 40 mg/kg (in vivo model)
Ren et al. [70]	2020	In vitro (AGS stomach cancer cell line)	↑GRP78 expression ↑p-eIF2α expression ↑CHOP expression	20 μM
Neal et al. [62]	2020	In vitro (retinal pigment cells treated with hydroquinone)	↑XBP1 expression ↑CHOP expression	15 and 30 μM
Arena et al. [78]	2021	In vitro (Her-2 positive breast cancer and salivary gland cancer cell lines)	↑CHOP expression	15 μM
Hecht et al. [79]	2021	In vivo (model of primary osteoarthritis in mice)	↓CHOP expression	0.25 g/L
Yu et al. [63]	2022	In vitro (tunicamycin-induced ER stress in chondrocytes)	↓CHOP expression	50 μM
Totonchi et al. [80]	2022	In vivo (mice liver-induced ischemia-reperfusion)	↓GRP78 expression ↓PERK expression ↓IRE1α expression ↓CHOP expression ↓XBP1 expression	0.02 and 0.2 mg/kg
Luo et al. [64]	2023	In vitro (bupivacaine-induced cytotoxicity in PC12 rat adrenal pheochromocytoma cells)	↓p-PERK expression ↓p-eIF2α expression ↓ATF4 expression	20 µM

PERK—protein kinase R (PKR)-like endoplasmic reticulum kinase; p-PERK—phosphorylated PERK; eIF2α—eukaryotic translation initiation factor 2α; p-eIF2α—phosphorylated eIF2α; IRE1—inositol-requiring enzyme 1; p-IRE1—phosphorylated IRE1; CHOP—C/EBP-homologous protein; XBP1—X-box binding protein 1; ATF4—activating transcription factor 4; ATF6—activating transcription factor 6; GRP78—glucose-regulated protein 78; GRP94—glucose-regulated protein 94; ERAD—endoplasmic reticulum-associated protein degradation; JNK—c-Jun N-terminal kinase. An downward arrow (↓) indicates a decrease, while a upward arrow (↑) indicates an increase.

**Table 2:** Influence of ER stress markers by cyanidin-3-o-glucoside, epicatechin, and chlorogenic acid.

Author(s)	Year	Experimental Model	ER Stress Modulating Substance	Altered Expression or Activity of ER stress Markers	Dosage
Thummayot et al. [81]	2016	In vitro (Aβ 25-35 induced neuronal cell death in SK-N-SH cells)	Cyanidin-3-o-glucoside	↓GRP78 expression ↓p-PERK expression ↓p-eIF2α expression ↓IRE1 expression ↓XBP1 expression ↓ATF6 expression ↓CHOP expression	0.2; 2; 18; and 20 µM
Chen et al. [82]	2022	In vitro (treated with palmitate isolated mouse pancreatic islets and INS-1E cells)	Cyanidin-3-o-glucoside	↓CHOP expression	12,5; 25; and 50 µM
Tu et al. [83]	2022	In vivo (induced periodontitis in rats)	Cyanidin-3-o-glucoside	↓CHOP expression ↓JNK and p-JNK expression	3 or 9 mg/kg
Peng et al. [84]	2022	In vitro (blue light-irradiated retinal pigment epithelial cells)	Cyanidin-3-o-glucoside	↓ATF4 expression ↓CHOP expression	10 and 25 μM
Bettaieb et al. [85]	2014	In vivo (high-fructose diet-fed rats)	Epicatechin	↓p-PERK expression ↓p-IRE1 expression ↓XBP1 splicing	20 mg/kg
Kang et al. [86]	2019	In vitro (methamphetamine-induced neurotoxicity in HT22 hippocampal neuronal cells)	Epicatechin	↓CHOP expression	10 and 20 μM
Ye et al. [87]	2016	In vivo (streptozotocin-induced diabetic nephropathy in rats)	Chlorogenic acid	↓CHOP expression ↓ATF6 expression ↓p-eIF2α expression ↓p-PERK expression	5 mg/kg, 10 mg/kg, 20 mg/kg
Wang et al. [88]	2017	In vivo (bleomycin-induced pulmonary fibrosis in mice), in vitro (pulmonary fibroblasts and RLE-6TN cells)	Chlorogenic acid	↓GRP78 expression ↓CHOP expression ↓p-PERK expression ↓ATF6 expression ↓caspases 9, 3, and 12 expression	15 mg/kg, 30 mg/kg, 60 mg/kg
Zhang et al. [89]	2018	In vitro (thapsigargin and palmitic acid-induced ER stress in rat hepatocytes)	Chlorogenic acid	↓GRP78 expression ↓GRP94 expression ↓CHOP expression	5 μmol/L
Kazaz et al. [90]	2022	In vivo (torsion/detorsion-induced testicular injury in rats)	Chlorogenic acid	↓GRP78 expression ↓ATF6 expression ↓CHOP expression	100 mg/kg
Rani et al. [91]	2022	In vitro (model of hyperglycemia in H9c2 embryonic rat heart cells)	Chlorogenic acid	↓p-PERK expression ↓p-eIF2α expression ↓ATF4 expression ↓p-IRE1 expression ↓TRAF2 expression ↓p-JNK expression ↓XBP1 expression ↓ATF6 expression	10 and 30 μM
Sari et al. [92]	2022	In vivo (diabetic model in rats)	Chlorogenic acid	↓GRP78 expression ↓XBP1 expression	12.5 mg/kg, 25 mg/kg, and 50 mg/kg
Moslehi et al. [93]	2023	In vivo (tunicamycin-induced ER stress in mice)	Chlorogenic acid	↓GRP78 expression ↓PERK expression ↓IRE1 expression ↓caspase 3 expression	20 and 50 mg/kg
Boonyong et al. [94]	2023	In vivo (indomethacin-induced gastrointestinal ulcer in rats)	Chlorogenic acid	↓p-PERK expression ↓p-eIF2α expression ↓ATF-4 expression ↓CHOP expression	100 mg/kg
Ping et al. [95]	2024	In vitro (isoproterenol stimulated H9c2 myocardial cells) In vivo (isoproterenol stimulated rats)	Chlorogenic acid	↓GRP78 expression ↓p-PERK expression ↓CHOP expression ↓caspases 12, 3, and 9 expression	90 mg/kg (in vivo model) 50 μM (in vitro model)

PERK—protein kinase R (PKR)-like endoplasmic reticulum kinase; p-PERK—phosphorylated PERK; eIF2α—eukaryotic translation initiation factor 2α; p-eIF2α—phosphorylated eIF2α; IRE1—inositol-requiring enzyme 1; p-IRE1—phosphorylated IRE1; CHOP—C/EBP-homologous protein; XBP1—X-box binding protein 1; ATF4—activating transcription factor 4; ATF6—activating transcription factor 6; GRP78—glucose-regulated protein 78; GRP94—glucose-regulated protein 94; TRAF 2 (TNF receptor-associated factor 2); JNK—c-Jun N-terminal kinase; p-JNK—phosphorylated JNK. An downward arrow (↓) indicates a decrease, while a upward arrow (↑) indicates an increase.

## Data Availability

Not applicable.
